# The role of arousal in the estimation of time‐to‐collision of threatening stimuli

**DOI:** 10.1002/pchj.762

**Published:** 2024-04-24

**Authors:** Caiwen Li, Yuming Xuan, Patrick Bruns, Xiaolan Fu

**Affiliations:** ^1^ State Key Laboratory of Brain and Cognitive Science Institute of Psychology, Chinese Academy of Sciences Beijing China; ^2^ Department of Psychology University of Chinese Academy of Sciences Beijing China; ^3^ Biological Psychology and Neuropsychology University of Hamburg Hamburg Germany

**Keywords:** arousal, threat, time‐to‐collision

## Abstract

The accurate estimation of time‐to‐collision (TTC) is essential for the survival of organisms. Previous studies have revealed that the emotional properties of approaching stimuli can influence the estimation of TTC, indicating that approaching threatening stimuli are perceived to collide with the observers earlier than they actually do, and earlier than non‐threatening stimuli. However, not only are threatening stimuli more negative in valence, but they also have higher arousal compared to non‐threatening stimuli. Up to now, the effect of arousal on TTC estimation remains unclear. In addition, inconsistent findings may result from the different experimental settings employed in previous studies. To investigate whether the underestimation of TTC is attributed to threat or high arousal, three experiments with the same settings were conducted. In Experiment 1, the underestimation of TTC estimation of threatening stimuli was replicated when arousal was not controlled, in comparison to non‐threatening stimuli. In Experiments 2 and 3, the underestimation effect of threatening stimuli disappeared when compared to positive stimuli with similar arousal. These findings suggest that being threatening alone is not sufficient to explain the underestimation effect, and arousal also plays a significant role in the TTC estimation of approaching stimuli. Further studies are required to validate the effect of arousal on TTC estimation, as no difference was observed in Experiment 3 between the estimated TTC of high and low arousal stimuli.

## INTRODUCTION

When driving on the highway, drivers need to accurately judge the movement of surrounding vehicles. When hitting an oncoming tennis ball, players need to accurately judge the flight of the tennis ball. In these scenarios, whether trying to avoid colliding with or to strike nearby objects, individuals need to estimate the time‐to‐collision (TTC) in real time. The TTC refers to the time from a certain moment until the object collides with the observer (DeLucia & Liddell, 1998; Field & Wann, 2005; Tresilian, 1999). It is also called time‐to‐contact in some other studies (Hecht & Savelsbergh, 2004; Smeets et al., 1996).

In the laboratory, researchers usually use the TTC judgment task to investigate the estimation of TTC with approaching objects (Brendel, 2019; Brendel et al., 2012; Hecht et al., 2021; Vagnoni et al., 2012, 2015, 2020). In this task, the object image is usually increased in size frame by frame on the monitor screen, which makes the object seem to move toward the participant. The approaching object will disappear after about 1 s. Then participants are asked to imagine that the disappeared object still continues to move toward them at the previous speed, and to quickly press a key when they feel the object collides with themselves. The reaction time is taken as the index of TTC estimation.

In general, the estimation of TTC mainly depends on physical characteristics such as on the distance between the object and the observer as well as the speed of the object (Regan & Gray, 2001; Wann, 1996), the change rate of the observer's visual angle (Kim, 2015), or the differential of *tau*, which is the ratio of visual angle subtended by the distance between any two points on the moving object divided by the rate of change of this angle (Battaglini & Ghiani, 2021; Hecht & Savelsbergh, 2004; Lee, 1976; Tresilian, 1997, 1999).

Recently, some studies have explored the effect of emotion on TTC estimation by investigating the TTC judgment of approaching threatening and non‐threatening stimuli (Brendel, 2019; Brendel et al., 2012; DeLucia et al., 2014; Vagnoni et al., 2012, 2015, 2017). The results of these studies often showed that threatening stimuli were more underestimated than non‐threatening ones.

For instance, Brendel et al. (2012) and DeLucia et al. (2014) used threatening pictures such as “a snarling pit bull” and “a biting snake” versus neutral or friendly pictures such as “lamp,” “plants and mushrooms” and “baby” from the International Affective Picture System (IAPS) to investigate the estimation of TTC. They found that the TTC estimation of threatening pictures are significantly shorter than that of neutral (Brendel et al., 2012) and friendly pictures (DeLucia et al., 2014). With a similar experimental paradigm, Vagnoni et al. (2012, 2015) also found that threatening animals (e.g., snakes and spiders) are judged to collide with observers earlier compared to non‐threatening animals (e.g., butterflies and rabbits). In Experiment 3 by Brendel et al. (2012), angry, happy, and neutral expressions were used as approaching stimuli in a TTC judgment task. The results showed that angry expressions shorten the TTC estimation compared with the other two kinds of expressions.

Although the above studies have found that the TTC of threatening stimuli is underestimated for various types of stimuli, there are also inconsistent results. For example, Brendel et al. (2014) found that threatening stimuli were not underestimated more than positive stimuli of high arousal. DeLucia et al. (2014) found that participants did not underestimate the TTC of angry compared with happy and neutral expressions, even though they used the same face models as Brendel et al. (2012) and a similar experimental procedure.

After careful comparison of Brendel et al. (2012) and DeLucia et al. (2014), differences in experimental settings can be found. These discrepancies may result in the inconsistency of the findings. Firstly, different presentation times of facial expressions were used. In Brendel et al. (2012), the presentation time was 200 and 800 ms, while it was 3000 ms in DeLucia et al. (2014). A large number of event‐related potential (ERP) studies have found that faces can induce a negative ERP component mainly in the visual cortex of the occipital lobe within a time window of 100–200 ms, which reaches its peak at about 170 ms (N170) and is considered to be a specific component of face processing (Batty & Taylor, 2003; Bentin et al., 1996; Itier & Taylor, 2004; Rossion, 2014; Rossion et al., 2000). A magnetoencephalography (MEG) study, which examined the role of presentation time in face perception, showed that the cortical neuromagnetic response induced by the face was strongest when the presentation time was 200 ms (Tanskanen et al., 2007). These findings suggested that the processing of faces can be completed in a short time window. Under the condition of long presentation time, participants' attention to emotional information of faces might, thus, be weakened, and participants may draw attention to other attributes of faces, such as gender, identity and skin texture (Fisher et al., 2016; Schweinberger & Soukup, 1998).

Secondly, different sizes of the presented faces were used in the two studies. The facial expressions used in Brendel et al. (2012) were presented on a 2.60 × 1.95 m projection screen, whose visual angle of the diagonal was 78 degrees at the distance of 2 m, while in DeLucia et al. (2014) the facial expressions were displayed on a 43 cm monitor, whose visual angle of the diagonal was 50 degrees at the distance of 45.72 cm. Previous studies have found that the size of the presented faces has an impact on the emotional arousal. Specifically, the arousal evaluation of a larger size face is significantly higher than that of a smaller one (Codispoti & de Cesarei, 2007). Therefore, differences in arousal between the two presentation conditions may have caused the discrepancy of TTC estimation of the same face.

Therefore, to eliminate the possible effect of experimental settings on TTC estimation, in the present study, all types of stimuli were presented in similar size and the same procedure was used in all experiments. Furthermore, our study addressed concerns about the role of arousal in TTC estimation. Threatening stimuli are not only more negative in valence, but also higher in arousal than neutral or non‐threatening stimuli. An important question arises therefore: is it valence or arousal that leads TTC to be underestimated?

Vagnoni et al. (2012) investigated this issue in a controlled experiment. They first displayed a natural threat or nonthreat animal image to participants for 1 s in a random order, and then a blue disk replaced the animal image and moved towards participants at different speeds and disappeared after 1 s. Participants were instructed to finish a TTC judgment task on the blue disk stimuli. The results showed that the estimation of TTC did not differ between the two priming conditions with the two types of static animal images. Nevertheless, in this controlled experiment, the approaching stimulus was a blue disk, whose arousal level was very different from an approaching snake or spider. Although the looming blue disk was primed by two types of images at the beginning of the trial, the individual's arousal level would decrease with time after the priming images disappeared. Therefore, the role of arousal cannot be effectively disentangled in this control experiment.

In comparison, the study of Brendel et al. (2014) seemed to be more effective. They used the same threat stimuli as in their 2012 study, and selected erotic and monetary pictures from the IAPS as high arousal positive stimuli. The results showed that there was no difference in TTC estimation between threatening and positive stimuli, suggesting that it is arousal but not valence that modulated the estimation of TTC of approaching objects. However, Brendel et al. did not control the arousal level of the two types of stimuli, as the arousal of threat stimuli was higher than that of positive stimuli in their experiment. In the present study the estimated TTC of positive stimuli and threatening stimuli would be compared with the arousal level being controlled.

### The present study

In the present study we tried to test the following two hypotheses in three experiments.

Threat Hypothesis The TTC of threatening stimuli is more underestimated than that of non‐threatening stimuli.

Arousal Hypothesis The TTC of stimuli with higher arousal is more underestimated than that with lower arousal.

In Experiment 1 the TTC estimation of threatening and non‐threatening stimuli was examined. Both the Threat Hypothesis and Arousal Hypothesis predict a difference in TTC estimation. In Experiment 2 the estimation of TTC of threatening and positive stimuli of similar arousal level was examined. A difference in TTC estimation is predicted by the Threat Hypothesis but not by the Arousal Hypothesis.

In Experiment 3 the estimated TTC of angry, neutral and happy faces were compared. Again, arousal of angry and happy faces was matched. The Threat Hypothesis would predict the TTC of angry faces to be more underestimated than that of neutral and happy faces, while the Arousal Hypothesis would predict that the TTC of angry and happy faces would be more underestimated than neutral faces.

## EXPERIMENT 1

In Experiment 1, the estimated TTC of threatening stimuli and non‐threatening ones was compared while their arousal was not controlled, in order to replicate previous findings of an underestimation of TTC for threatening versus non‐threatening stimuli (Vagnoni et al., 2012) with our experimental setup.

### Method

#### Participants

Twenty undergraduate and graduate students (*M*_age_ = 22.40 years, *SD*_age_ = 2.35 years, 11 female) volunteered to take part in the experiment. To achieve a power of 1‐*β* = 0.8 at a significance level of *α* = 0.05, the sample size was determined by conducting a power analysis in G*Power 3.1.9.7 (Faul et al., 2007) based on an average effect size of $\eta_{p}^{2}$ = 0.26 from previous studies (Brendel et al., 2012; DeLucia et al., 2014; Vagnoni et al., 2017). The power analysis showed that at least 14 participants would be required. All participants reported normal or corrected‐to‐normal vision. Informed consent was obtained from each participant prior to the experiment. The experiment procedures were approved by the Ethics Committee of the Institute of Psychology, Chinese Academy of Sciences. After finishing the experiment, participants were paid 35–45 RMB to compensate for their time.

#### Materials

**FIGURE 1:**
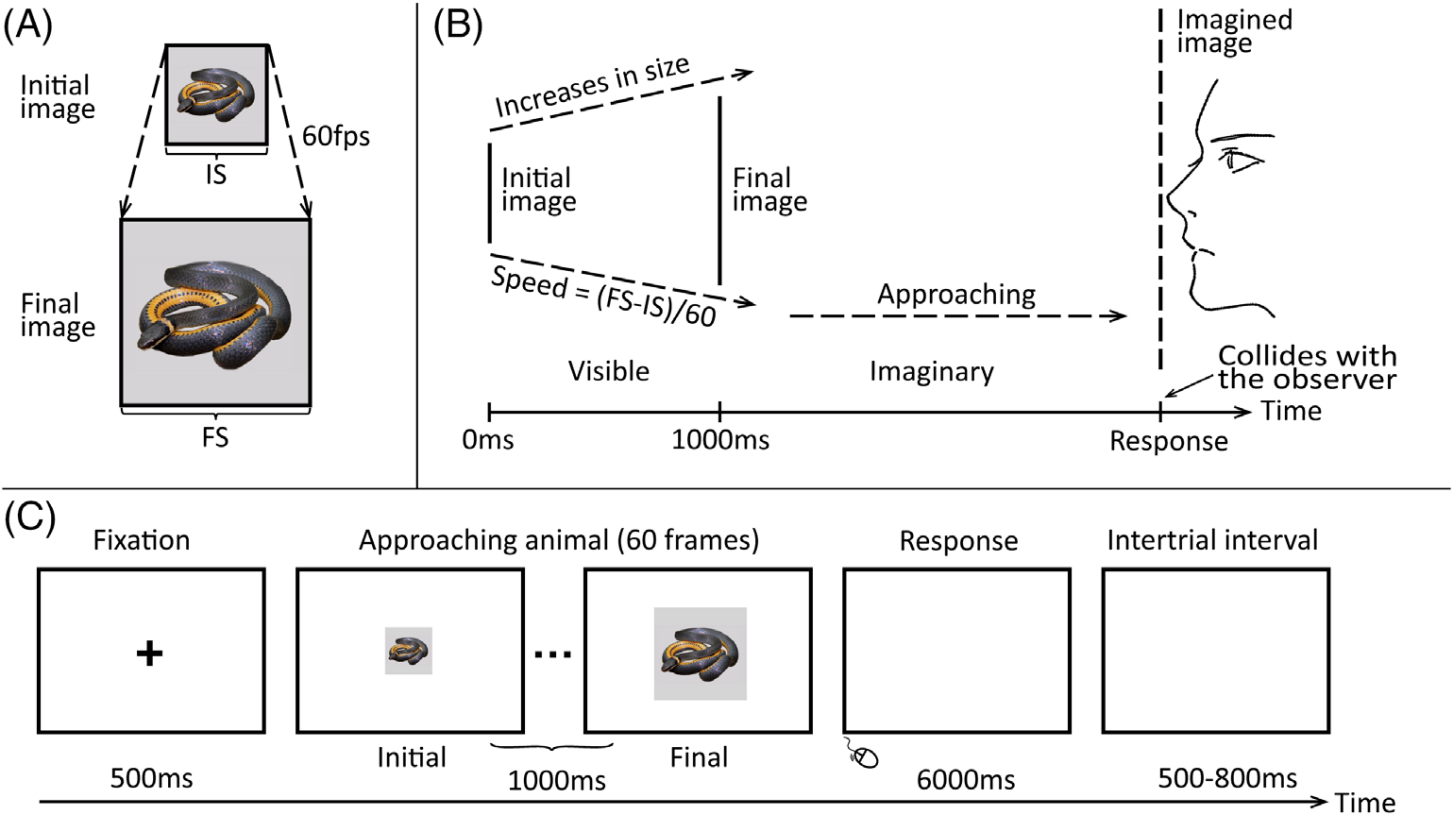
Schematic illustrations of the simulated approaching movement, time‐to‐collision (TTC) manipulation and the trial structure in the present study. (A) The approaching movement was simulated by enlarging the size of the image from initial frame (IS) to final frame (FS) in 60 frames over 1 s. The central points of all frames were set at the center of the screen. (B) The observers would see an image increasing in size gradually at the beginning of a trial, then the image would disappear after 1000 ms. The observers were instructed to imagine that the image was still moving toward them at the previous speed after it disappeared, and to press the left mouse button quickly at the moment when they felt it would have collided with them. (C) The whole procedure of a trial under threatening condition in Experiment 1.

A total of 160 animal pictures taken from Vagnoni et al. (2012) served as stimuli. The pictures were assigned to threatening (snakes and spiders) and non‐threatening (butterflies and rabbits) stimuli, 80 images for each of the two categories. All of them were in color, with only a single object in content, and the background was gray (RGB: 213, 211, 213). Every image was converted into an animation (.AVI) with approaching movement by enlarging the size from small to large in 60 frames over 1 s (Figure 1A) in MATLAB (MathWorks, Natick, MA) using custom scripts. Each image was set to two initial frame sizes (16.20° × 16.20° and 20.20° × 20.20°) to ensure that participants could not make responses based on the final size of the stimuli only. The final size means the size of the final frame of the image before it disappears. The two kinds of images (threatening vs. non‐threatening) were presented under five actual TTC conditions: 3.0, 3.5, 4.0, 4.5, and 5.0 s. The five actual TTC conditions were manipulated by changing the final frame size of the images using the following formula given by Vagnoni et al. (2012):

$$\mathrm{FS}=\frac{\text{IS}}{1-\frac{D}{\mathrm{TTC}}}$$

In the formula, FS and IS indicate the final and initial frame size in pixel, D refers to display duration (1 s in all experiments in the present study), and TTC denotes actual TTC conditions. For instance, given that the initial size of the image is 16.20° × 16.20°, actual TTC is 3.0 s, and D is 1 s, the final size of the image should be 24.30° × 24.30°.

All images were rated on a 9‐point Likert scale in terms of valence and arousal by the participants after completing the main task of Experiment 1.

#### Apparatus and procedure

The stimuli were presented on a 24.5‐inch monitor with a resolution of 1920 × 1080 pixels and a refresh rate of 60 Hz. Stimulus presentation and data collection were controlled by E‐Prime 2.0 (Psychology Software Tools, Inc., Pittsburgh, PA).

The experiment was conducted in a dimly lit and sound‐insulated room. Participants were seated in front of the monitor, and their heads were placed on a chin rest at a distance of 40 cm. They were given both written and oral task instructions. Figure 1 depicts a schematic illustration of the trial structure in the TTC judgment task. A black cross was presented in the center of the screen for 500 ms at the beginning of the trial. Then an animation of a picture with approaching movement was displayed at the same location as the cross, and disappeared after 1000 ms. Participants were instructed to imagine that the approaching picture still moved toward them at the previous speed after it disappeared and to press the left mouse button quickly at the moment when they felt it would have collided with them. They were asked to respond within 6000 ms. The next trial began after a random intertrial interval of 500–800 ms. Before the main experimental session, participants completed 20 practice trials. All trials (consisting of two types of images with five actual TTC conditions) were presented in a randomized order and were divided into eight blocks. Participants were allowed to take a short rest between each block. It took approximately 50 min to finish the experiment.

### Data analysis

Trials in which participants did not respond or in which the reaction time (RT) exceeded 3 standard deviations from the mean RT were excluded before the data analysis. A repeated measures ANOVA was conducted to analyze mean RTs of TTC estimation using SPSS software (Version 22.0, IBM Analytics, Armonk, NY). The *p‐*values for multiple comparisons were adjusted by Bonferroni correction.

In addition, we also conducted Bayesian repeated measures ANOVA using JASP software (0.18.0 Version, JASP Team, Amsterdam). Bayes factors *BF*_10_ are reported for comparing the model including the effect of interest to the null model. We interpreted Bayes factors of 1–3 as anecdotal, 3–10 as moderate, 10–30 as strong, 30–100 as very strong, and >100 as extreme evidence (Wagenmakers et al., 2018). Accordingly, if *BF*_10_ was less than 0.33 (or 0.1), it would show that the null hypothesis is supported by moderate or strong evidence. If *BF*_10_ falls between 0.33 and 1, we would interpret that there was no significant difference and Bayesian analysis supported the null rather than the alternative hypothesis, albeit only weakly.

### Results

For the ratings of stimuli, a paired sample *t‐*test showed that the mean rating of valence of threatening stimuli (*M* = 2.35, *SD* = 0.54) was significantly lower than for non‐threatening stimuli (*M* = 5.30, *SD* = 0.85), *t* (19) = 14.822, *p* < .001, *d* = 3.31, *BF*_10_ = 2.285 × 10^9^. It also showed that the mean rating of arousal of threatening stimuli (*M* = 5.81, *SD* = 1.47) was significantly higher than for non‐threatening stimuli (*M* = 5.06, *SD* = 0.95), *t* (19) = 2.424, *p* = .025, *d* = 0.54, *BF*_10_ = 4.671. The statistics of the stimuli evaluation are summarized in Table 1.

**TABLE 1:** The mean ratings of the valence and arousal of the stimuli in all experiments.

Experiments	Stimulus type	Valence (*M* ± *SD*)	Arousal (*M* ± *SD*)
Experiment 1	Threatening	2.35 ± 0.54	5.81 ± 1.47
	Non‐threatening	5.30 ± 0.85	5.06 ± 0.95
Experiment 2	Threatening	2.50 ± 0.79	5.32 ± 1.73
	Positive	6.74 ± 0.85	6.15 ± 0.83
Experiment 3	Angry	2.44 ± 0.94	5.35 ± 1.68
	Happy	6.86 ± 0.81	5.36 ± 1.35
	Neutral	4.64 ± 0.49	3.32 ± 1.42

*Note*: Ratings of valence and arousal in Experiment 1 were done by the same participants who did the TTC task, but in Experiments 2 and 3 were done by another group of participants different from the TTC task.

Abbreviation: TTC, time‐to‐collision.

**FIGURE 2:**
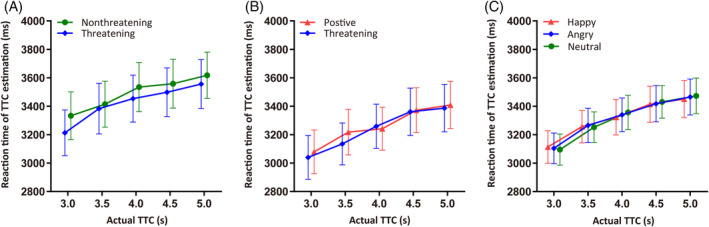
Mean reaction times of time‐to‐collision (TTC) estimation of different types of stimuli in Experiments 1, 2 and 3. The TTC estimation of threatening and non‐threatening pictures in Experiment 1 (A), of threatening and positive pictures in Experiment 2 (B), and of three types of facial expressions in Experiment 3 (C). Error bars represent standard errors of the means.

Trials (1.72%) in which participants did not respond or in which the reaction time (RT) exceeded 3 standard deviations from the mean RT were excluded. A 2 (stimulus type: threatening vs. non‐threatening) × 5 (actual TTC: 3.0, 3.5, 4.0, 4.5, and 5.0 s) repeated measures ANOVA showed that the main effect of stimulus type was significant, *F* (1, 19) = 10.071, *p* = .005, $\eta_{p}^{2}$ = 0.346, *BF*_10_ = 5.957. Mean RT of TTC estimation were shorter for threatening (*M* = 3430.815 ms, *SE* = 166.756, 95% confidence interval [CI] = [3081.790, 3779.839]) than for non‐threatening (*M* = 3500.349 ms, *SE* = 164.291, 95% CI = [3156.483, 3844.214]) animals. The main effect of actual TTC was also significant, *F* (4, 76) = 16.313, *p* < .001, $\eta_{p}^{2}$= 0.462, *BF*_10_ = 1.075 × 10^7^. The mean RT of TTC estimation was shorter in the 3 s actual TTC condition than in the other four actual TTC conditions, all *p*s ≤ .027. In the other pairwise comparisons, there were significant differences except for pairwise comparisons of 4.0 and 4.5 s, 4.5 and 5.0 s actual TTC conditions, all *p*s ≤ .019 for the significant and all *p*s ≥ .188 for the non‐significant ones. The interaction effect between stimulus type and actual TTC was not significant, *F* (4, 76) = 0.855, *p* = .495, $\eta_{p}^{2}$ = 0.043, *BF*_10_ = 0.118. The results are shown in Figure 2A.

To investigate the effect of approaching movement on TTC estimation, we conducted one sample *t*‐test to compare mean RTs of TTC estimation with actual TTC under five TTC conditions separately. The results showed that the mean RTs of TTC estimation were shorter than actual TTCs under 4.0, 4.5, and 5 s conditions, *t*s ≤ −2.937, *p*s ≤ .004, *BF*_10_ ≥ 11.704; the mean RTs of TTC estimation were not significantly different from actual TTCs under 3.0 and 3.5 s conditions, *t*s ≥ −0.547, *p*s ≥ .295, *BF*_10_ ≤ 0.370.

### Discussion

In Experiment 1, the TTC of threatening stimuli was underestimated compared with that of non‐threatening stimuli. The result was in line with the previous studies (Brendel, 2019; Brendel et al., 2012; Vagnoni et al., 2012, 2015). It indicated that individuals often make more conservative estimations of TTC when faced with approaching threatening objects in their surroundings. As proposed by Vagnoni et al. (2012), the underestimation of TTC may serve an adaptive role in leaving additional time for either fight or flight when facing threats.

## EXPERIMENT 2

In Experiment 2, the estimated TTC of threatening and positive stimuli of the same arousal level were compared.

### Method

#### Participants

Twenty new students (*M*_age_ = 22.55 years, *SD*_age_ = 1.76 years, 10 female) took part in Experiment 2. Before the experiment, informed consent was obtained from all the participants. The sample size was estimated in the same way as in Experiment 1.

#### Materials

The threatening stimuli were the same as in Experiment 1. The positive stimuli were 80 pictures collected from the internet, which consisted of two categories: food and banknotes, 40 for each category. Backgrounds of all positive pictures were replaced with gray. The approaching movement of positive stimuli was simulated in the same way as in Experiment 1. A group of 11 participants who did not participate in the main task rated the valence and arousal of all images in Experiment 2.

#### Apparatus and procedure

The apparatus and the procedure were the same as in Experiment 1, except that the stimuli were threatening and positive as described above.

### Results

The results of a paired sample *t‐*test showed that the ratings of the valance of the threatening stimuli (*M* = 2.50, *SD* = 0.79) were significantly lower than those of the positive stimuli (*M* = 6.74, *SD* = 0.85), *t* (10) = 9.949, *p* < .001, *d* = 3.00, *BF*_10_ = 2.050 × 10^4^. There was no significant difference in the ratings of arousal between the threatening (*M* = 5.32, *SD* = 1.73) and the positive stimuli (*M* = 6.15, *SD* = 0.83), *t* (10) = 1.311, *p* = .219, *d* = 0.40, *BF*_10_ = 0.592. The statistics of the stimuli evaluation are summarized in Table 1.

A 2 (stimulus type: threatening vs. positive) × 5 (actual TTC: 3.0, 3.5, 4.0, 4.5, and 5.0 s) repeated measures ANOVA showed that the main effect of stimulus type was not significant, *F* (1, 19) = 1.301, *p* = .268, $\eta_{p}^{2}$ = 0.064, *BF*_10_ = 0.402. The main effect of actual TTC was significant, *F* (4, 76) = 23.231, *p* < .001, $\eta_{p}^{2}$= 0.550, *BF*_10_ = 6.897 × 10^9^. The mean RT of TTC estimation was shorter in the 3 s actual TTC condition than in the other four actual TTC conditions (all *p*s ≤ .003). In other pairwise comparisons, there were significant differences except for comparisons of 3.5 and 4.0 s, 4.5 and 5.0 s actual TTC conditions, all *p*s ≤ .022 for the significant and all *p*s ≥ .164 for the non‐significant ones. The interaction effect between stimulus type and actual TTC was not significant, *F* (4, 76) = 1.557, *p* = .194, $\eta_{p}^{2}$ = 0.076, *BF*_10_ = 0.328. The results are shown in Figure 2B.

Similarly, one sample *t*‐test was conducted to test the difference between the mean RT of TTC estimation and actual TTC under each TTC condition. It showed that the mean RTs of TTC estimation were shorter than the actual TTCs under 3.5, 4.0, 4.5, and 5 s conditions, *t*s ≤ −2.076, *p*s ≤ .026, *BF*_10_ ≥ 2.622, but not under 3.0 s condition, *t* = 0.382, *p* = .647, *BF*_10_ = 0.179.

### Discussion

The results of Experiment 2 showed that the TTC of threatening stimuli was not underestimated compared with that of positive stimuli. These findings are consistent with Brendel et al. (2014) and cannot be explained by the Threat Hypothesis but are in line with the Arousal Hypothesis. Given that the positive and the threatening stimuli had the same arousal level, there should be no difference in estimated TTC of these two types of stimuli according to the Arousal Hypothesis. It should be noted that the results of Bayesian factor analysis indicated that the data of Experiment 2 had a relatively weak support for the null hypothesis.

## EXPERIMENT 3

There was not a condition of neutral stimuli in Experiment 2 and, thus, we were not able to compare the estimated TTC of emotional stimuli to neutral ones like in Experiment 1. To directly compare all three valence levels, in Experiment 3, we used angry, happy, and neutral faces as stimuli. Threatening angry faces and positive happy faces were of the same arousal level as in Experiment 2. Besides, the stimuli used in Experiments 1 and 2 were not matched for low‐level characteristics, such as luminance, color, contrast and so forth. Experiment 3 was designed to control for these potential confounding factors. The images of different facial expressions used in Experiment 3 were taken from the same models and, thus, their contrasts were well matched.

### Method

#### Participants

Twenty new undergraduate and graduate students (14 female, *M*_age_ = 22.40 years, *SD*_age_ = 2.35 years) participated in the experiment after providing informed consent.

#### Materials

Faces of seven female and seven male models from the NimStim Set (Tottenham et al., 2009) were used as stimuli. The numbers of the models in the NimStim Set were 3, 7, 8, 9, 10, 11, 18, 20, 23, 34, 37, 41, 42, and 45. Angry, happy, and neutral emotional faces of these models were used in this experiment. The background of all facial expressions was white (RGB: 255, 255, 255). We calculated the contrast of each face using a custom script in MATLAB. A one‐way ANOVA showed that there was no difference in contrast among the three kinds of expressions, *F* (2, 87) = 1.81, *p* = .170, $\eta_{p}^{2}$ = 0.04, *BF*_10_ = 0.414. We converted each of these faces into animations of approaching movement in the same manner as in Experiments 1 and 2. All faces were rated on the dimensions of valence and arousal on a 9‐point Likert scale by 10 participants who did not take part in the main task of Experiment 3.

#### Apparatus and procedure

The apparatus and procedure were the same as in Experiments 1 and 2.

### Results

The results of ratings were analyzed by a one‐way ANOVA and post hoc comparisons with Bonferroni correction. The main effect of emotional category of facial expressions was significant in valence, *F* (2, 29) = 82.875, *p* < .001, $\eta_{p}^{2}$ = 0.86, *BF*_10_ = 1.827 × 10^9^. The mean ratings of valence for angry (*M* = 2.44, *SD* = 0.94) and neutral (*M* = 4.64, *SD* = 0.49) faces were significantly lower than those for happy (*M* = 6.86, *SD* = 0.81) faces, and ratings for angry faces were significantly lower than those for neural faces (all *p*s < .001, *d*s ≥2.90, *BF*_10_ ≥ 3.385 × 10^3^). The main effect of emotional category was also significant in arousal, *F* (2, 29) = 6.24, *p* = .006, $\eta_{p}^{2}$ = 0.32, *BF*_10_ = 8.337. The mean ratings of arousal for angry (*M* = 5.35, *SD* = 1.68) and happy faces (*M* = 5.36, *SD* = 1.35) were significantly higher than those for neutral (*M* = 3.32, *SD* = 1.42) faces (both *p*s < .05, *d*s ≥1.36, *BF*_10_ ≥ 5.587), while there was no significant difference in ratings of arousal between angry and happy faces (*p* > .999, *d* = 0.002, *BF*_10_ = 0.397). The mean ratings of the valence and arousal of the stimuli in Experiment 3 are shown in Table 1.

Trials (1.33%) in which participants did not respond or in which RTs exceeded 3 standard deviations from mean RTs were eliminated from further analysis. Mean RTs of TTC estimation for the three kinds of facial expressions were analyzed by a 3 (facial emotion: angry vs. happy vs. neutral) × 5 (actual TTC) repeated‐measures ANOVA. The results are depicted in Figure 2C. The main effect of facial emotion was not significant, *F* (2, 38) = 0.061, *p* = .941, $\eta_{p}^{2}$ = 0.003, *BF*_10_ = 0.065. The main effect of actual TTC was significant, *F* (4, 76) = 15.406, *p* < .001, $\eta_{p}^{2}$ = 0.448, *BF*_10_ = 4.663 × 10^6^. Simple effect test showed that the estimation of TTC for the 3.0 s actual TTC condition was significantly shorter than for the other four actual TTC conditions (all *p*s ≤ .041). In other pairwise comparisons, there were significant differences except for the comparison of 3.5 and 4.0 s, 4.0 and 4.5 s, 4.5 and 5.0 s actual TTC conditions, all *p*s ≤ .002 for the significant ones and *p*s ≥ .053 for the non‐significant ones. The interaction effect of facial emotion and actual TTC was not significant, *F* (8,152) = 0.106, *p* = .999, $\eta_{p}^{2}$ = 0.006, *BF*_10_ = 0.008.

Furthermore, the role of approaching movement in TTC estimation of faces was also examined with one sample *t*‐test under each TTC condition. It showed that the mean RTs of TTC estimation were shorter than actual TTCs under 3.5, 4.0, 4.5, and 5 s conditions, *t*s ≤ −2.155, *p*s ≤ .022, *BF*_10_ ≥ 2.974, but not under 3.0 s condition, *t* = 0.983, *p* = .831, *BF*_10_ = 0.129.

### Discussion

In Experiment 3 there were no differences in the estimated TTC of three types of facial expressions. These results were not consistent with Experiments 1 and 2, because they could be predicted neither by the Threat Hypothesis nor by the Arousal Hypothesis. The Threat Hypothesis would predict the TTC of angry faces to be more underestimated than that of neutral and happy faces. The Arousal Hypothesis would predict that the TTC of angry and happy faces would be more underestimated than neutral faces. The finding of no difference between the estimated TTC of angry and happy faces was consistent with the Arousal Hypothesis and the results of Experiment 2.

These findings are, however, consistent with those of DeLucia et al. (2014), but inconsistent with those of Brendel et al. (2012). DeLucia et al. (2014) suggested that the duration of approaching facial expressions in their experiment was too long (3 s) so that subjects adapted to these social threatening stimuli and hence did not underestimate the TTC. The duration of approaching facial expressions in the present experiment was set to 1 s, which was similar to the presentation time in Brendel et al. (2012), but we still did not find TTC underestimation of facial threatening stimuli. Furthermore, since we used the same duration in all three experiments, the inconsistency of the experimental results could not be explained by the variation of the duration.

In Experiment 3, the subjects who evaluated the stimuli did not participate in the main task, and therefore their results may not be representative of the other group. However, it seems unlikely that the null effect of arousal in Experiment 3 is due to potential differences in ratings in the observers who performed the main task as our results agree well with ratings in other studies in which NimStim faces were used (e.g., Min & Kim, 2022; Smith et al., 2013). For example, in Study 2 of Smith et al. (2013), the results of facial expression evaluations showed higher arousal levels for anger and happy expressions compared to neutral expressions in accordance with our rating results.

One possible explanation for the null effect of facial expression on TTC estimation is that the early stage (and not the late stage) of information processing is more likely to be affected by facial expression in the situation of approaching or looming movement. Yu et al. (2022) explored the time course of the neural processing of approaching threatening facial expressions using ERP techniques. They found that the P1 was enhanced by looming angry faces compared to neutral faces. Some studies suggested that P1 modulated by emotions are believed to be associated with global processing of low‐level visual features of affective information or enhanced attention to the global features of these stimuli (Batty & Taylor, 2003; Pourtois et al., 2004; Schindler et al., 2021). The findings suggested that the influence of facial emotion on approaching movement may take place at the very early stages of visual processing—the sensory or perceptual processing of approaching stimuli. However, we tend to view the estimation of TTC as late‐stage information processing—a “post‐perceptual cognitive processing (e.g., cognitive extrapolation of motion after the object disappeared)” as supposed by DeLucia et al. (2014). The effect of facial emotion on TTC may thus be weakened by late cognitive processes, such as cognitive reappraisal.

## GENERAL DISCUSSION

In the present study we tested the Threat Hypothesis and the Arousal Hypothesis in three experiments using the same procedure with the same experimental settings. The Threat Hypothesis predicts that the TTC of threatening stimuli will be underestimated compared to non‐threatening stimuli, while the Arousal Hypothesis predicts that the TTC of stimuli with higher arousal will be underestimated compared to that with lower arousal. The results of the three experiments are summarized in Table 2.

**TABLE 2:** Summary of the results of the three experiments.

Experiments	Stimuli types	Arousal	Valence	Estimated TTC	Threat H	Arousal H
Experiment 1	T, N	T > N	T < N	T < N	✓	✓
Experiment 2	T, P	T = P	T < P	T = P	×	✓
Experiment 3	T, N, P	T = P > N	T < N < P	T = P = N	×	☑

*Note*: T: threatening, N: neutral, P: positive, Threat H: Threat Hypothesis, Arousal H: Arousal Hypothesis, ✓: consistent with the hypothesis, ×: inconsistent with the hypothesis, ☑: partly consistent with the hypothesis.

Across three experiments, the Arousal Hypothesis seems to better explain our results than the Threat Hypothesis. As shown in Table 2, only the results of Experiment 1 are consistent with the Threat Hypothesis, while the results of Experiments 1 and 2 and part of the results of Experiment 3 are consistent with the Arousal Hypothesis. Therefore, our results indicate that arousal may play an important role in the estimation of TTC. These findings are consistent with previous studies reporting an influence of emotional arousal of stimuli on time perception (Droit‐Volet, 2013; Droit‐Volet et al., 2004; Gil & Droit‐Volet, 2012; Min & Kim, 2022).

The Scalar Expectancy Theory (Gibbon et al., 1984; Lake et al., 2016; Lehockey et al., 2018; Treisman, 1963) is a prominent theory used to explain the role of arousal in time perception. The theory posits that time perception involves three main stages: clock, memory, and decision. During the clock stage, a pacemaker emits pulses, which are then collected by an accumulator. The number of pulses represents the length of time. It has been suggested that the effect of arousal on time perception is to increase the pulse frequency of the pacemaker, that is, to accelerate the internal clock, so that the number of pulses collected by the accumulator also increases (Cheng et al., 2016; Droit‐Volet, 2013; Droit‐Volet et al., 2015; Droit‐Volet & Gil, 2016; Lake et al., 2016; Ogden et al., 2019), which leads to an overestimation of time.

There are two types of tasks of time estimation when considering the frequency of an internal clock. For simplicity, we refer to them as type‐I and type‐II tasks, and we suppose that the mechanism of the internal clock is metaphorically referred to as counting. In a type‐I task, participants are required to estimate the duration of an event (e.g., 1 min). Thus, the duration of the event becomes the actual reference for the participants. If the task is to be completed by counting from 1 in 1 min (the actual duration of the event), the faster the frequency of counting, the larger the number of pulses and hence the longer the estimated time. This is the case of overestimation. For example, Ogden et al. (2019) asked participants to judge the duration of images presented on the computer screen for certain durations. They found that the participants overestimated the duration of negative images compared to neutral images.

In a type‐II task, participants need to estimate the duration without an actual reference. If the task is to be done by counting to a certain number, then the faster the frequency of counting, the shorter the estimated time. This is the case of underestimation. For example, if we are to estimate 1 min by counting from 1 to 60, the faster the frequency of counting, the shorter the estimated “1 min” will be. In TTC estimation, the participants are to estimate when the perceived approaching image will collide with themselves. They are not given a specific duration of time to do the task. Thus, this is a type‐II task which is likely to result in underestimation. Sarigiannidis et al. (2020) found that participants underestimate the duration of time under a fear condition in a temporal bisection task. Zhao and Zeng (2022) also found that participants reproduced shorter durations under electrical stimulation compared to non‐electrical stimulation in a time‐reproduction task. In these two studies, there was not an actual duration of time for the participants to compare with when completing the experimental tasks, that is, they performed a type‐II task.

Regarding the impact of clock speed on TTC estimation, we assume that there are different clock speeds between high and low arousal conditions, but we do not predict a difference of clock speed between the early (1 s of stimuli presentation) and late stage (TTC estimation) of each trial. In our experiments, the subjects did not know that the duration of the presented looming stimuli was 1 s. At the beginning of each trial, the clock speed was induced by the looming stimuli and it might keep constant to the end of this trial. However, there could be a difference in clock speed between trials because of the differences of arousal of the stimuli between trials.

In addition, the results of three experiments showed that participants underestimated the TTC in four actual TTC conditions (3.5, 4.0, 4.5, and 5.0 s; but not 3.5 s in Experiment 1). This phenomenon may be due to the approaching movement of the objects. Some studies found that looming stimuli (e.g., dots, disks, arrows, and gratings) receive priority in information processing (Malek et al., 2012; Parker & Alais, 2007; Tyll et al., 2013; von Muhlenen & Lleras, 2007), because looming is a warning signal, containing aggressive and offensive information from unknown objects, and hence can cause fear responses (Schiff et al., 1962). Therefore, a conservative estimation of TTC of approaching stimuli is an efficient way to protect one's own safety, but why was TTC not underestimated in the 3.0 s (and 3.5 s in Experiment 1) condition? Previous studies found a simple range effect in TTC estimation, suggesting that participants would be biased toward the average duration of stimuli in the TTC judgment task (Battaglini & Ghiani, 2021; Bennett et al., 2010). In the range of durations (3.0–5.0 s) used in our experiments, the 3.0 s (and 3.5 s) condition is on the lower side of the average duration. Therefore, it is more likely that participants overestimate the TTC in the 3.0 s actual TTC condition based on the simple range effect, which would counteract any general underestimation effects to looming stimuli.

Furthermore, the results showed a difference of less than 400 ms of estimated TTC when the difference of actual TTC was 2 s (the actual TTC varied from 3 to 5 s). This is the case in the present study as well as in previous studies. For example, the difference of estimated TTC was about 1000 ms in Vagnoni et al. (2012) and about 700 ms in Vagnoni et al. (2015) although the difference of actual TTC was also 2 s in their studies. This may be due to the fact that the physical and psychological quantity of a stimulus are not equivalent. For time estimation and other tasks, people are good at making relative judgments rather than absolute ones. As shown in Figure 2, the estimated TTC increased with the increase of actual TTC. This means that participants were able to distinguish between long duration and short duration but could not tell the absolute value of the time difference or of the certain duration of time.

## CONCLUSIONS AND OPEN QUESTIONS

In the current study, the mechanism underlying the underestimation of TTC of emotional stimuli was investigated. Our results demonstrated that the estimation of TTC of approaching stimuli is more dependent on arousal than on threat.

There are some remaining issues to be addressed in future studies. The first is the control of the properties of stimuli. For emotional properties, in Experiments 2 and 3, we compared the estimated TTC of the stimuli of the same arousal but of different valence. Valuable results may be obtained by comparing the TTC estimation of stimuli of the same valence but of different arousal.

Besides, in all three experiments, low‐level characteristics of the images were not matched, such as luminance and/or contrast. However, in Experiment 3, happy, angry, and neutral faces of the same identities were used, which should have largely reduced differences of physical properties of different types of stimuli. Nevertheless, future studies should systematically assess the influence of low‐level stimulus characteristics on TTC estimation.

Secondly, the ratings of valence and arousal were obtained from static images in our experiments, while the images in the TTC judgment task were approaching observers and the arousal of the approaching stimuli may vary with the change in distance to the observers. Psychophysiological measures, such as skin conductance level, might be useful to evaluate the varying arousal of the stimuli during the looming procedure.

Finally, as in previous studies, approaching movement of emotional stimuli in this study was simulated by enlarging the size of the images frame by frame on the monitor. This is not equivalent to what we see in real life. With the advantage of being more realistic in changes of distance, motion, and depth (Rolin et al., 2019; Scarfe & Glennerster, 2015), virtual reality technology may compensate for this deficiency in future studies.

## CONFLICT OF INTEREST STATEMENT

The authors declare no conflicts of interest.

## ETHICS STATEMENT

This study was approved by the Ethics Committee of the Institute of Psychology, Chinese Academy of Sciences. Informed consent was obtained from each participant prior to the experiment.

## Data Availability

The data that support the findings of this study are available from the corresponding author upon reasonable request.
